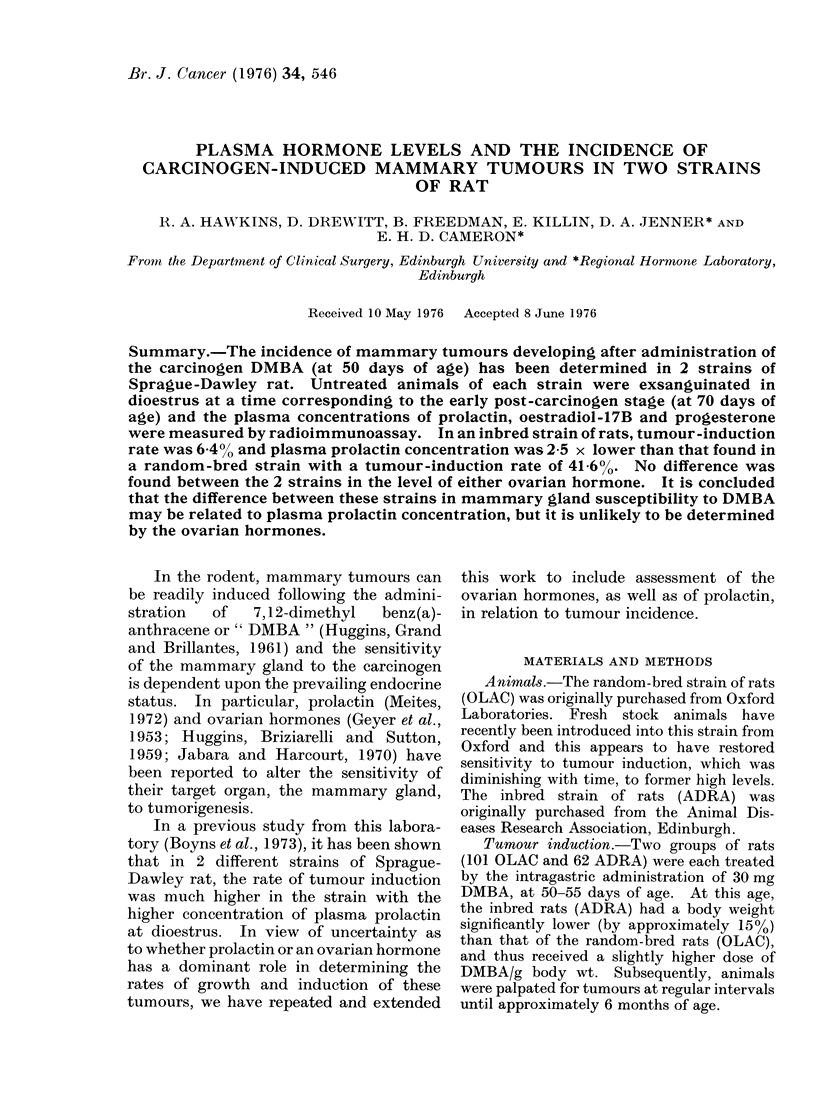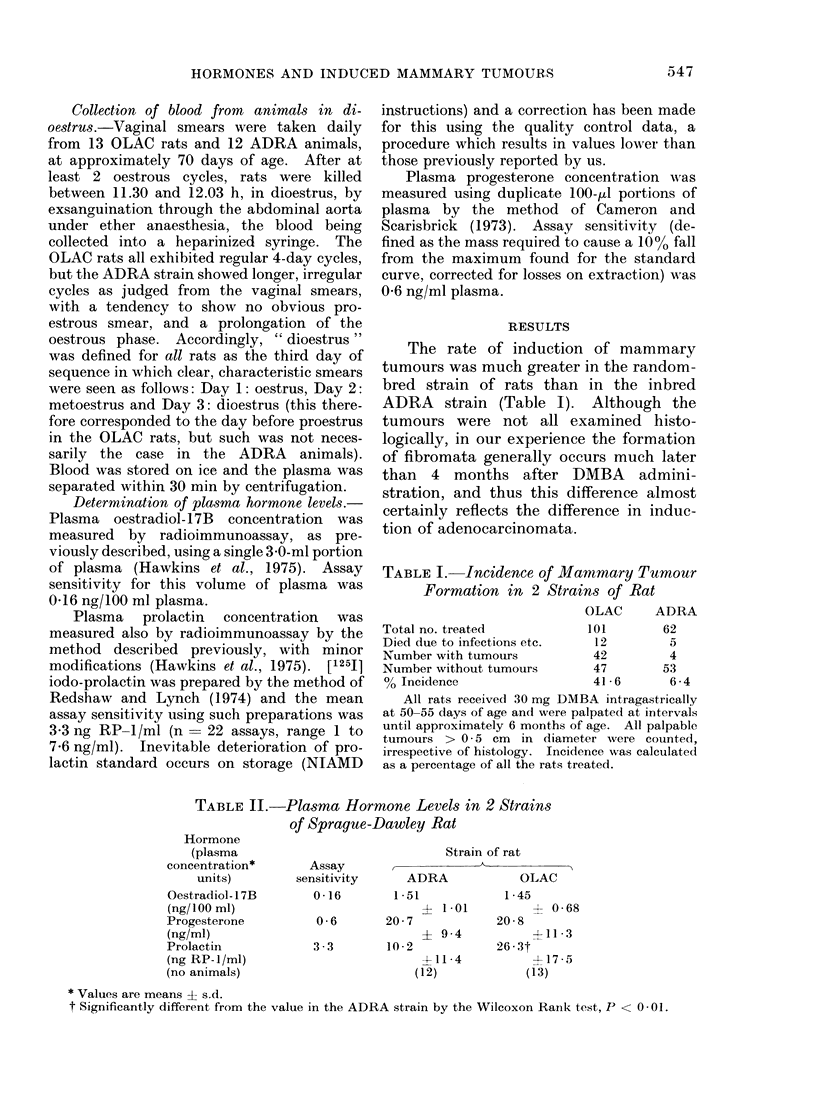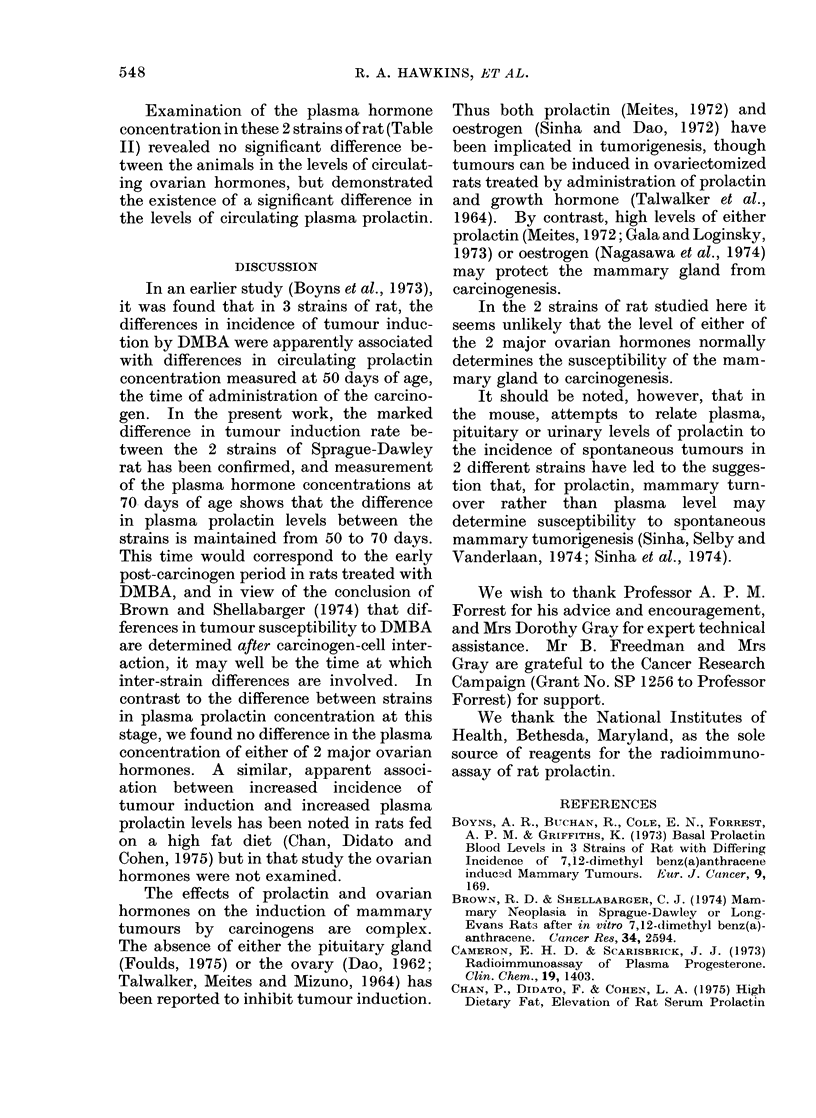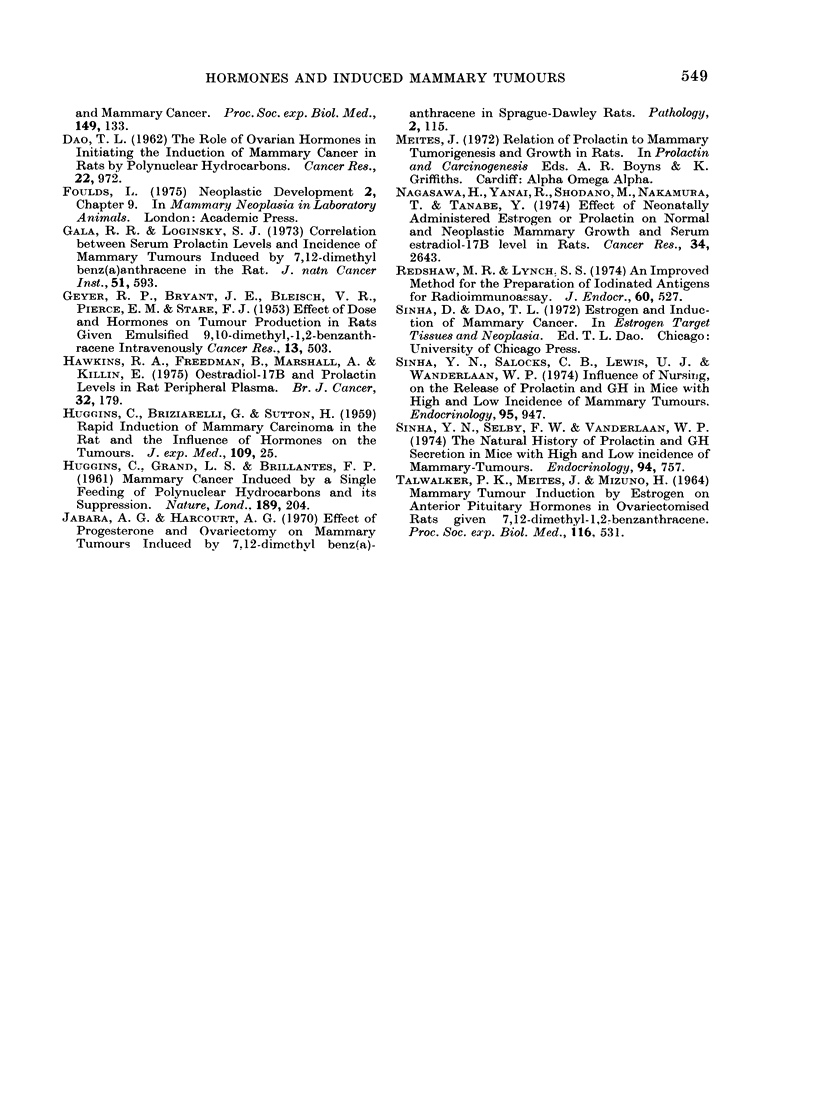# Plasma hormone levels and the incidence of carcinogen-induced mammary tumours in two strains of rat.

**DOI:** 10.1038/bjc.1976.209

**Published:** 1976-11

**Authors:** R. A. Hawkins, D. Drewitt, B. Freedman, E. Killin, D. A. Jenner, E. H. Cameron

## Abstract

The incidence of mammary tumours developing after administration of the carcinogen DMBA (at 50 days of age) has been determined in 2 strains of Sprague-Dawley rat. Untreated animals of each strain were exsanguinated in dioestrus at a time corresponding to the early post-carcinogen stage (at 70 days of age) and the plasma concentrations of prolactin, oestradiol-17B and progesterone were measured by radioimmunoassay. In an inbred strain of rats, tumour-induction rate was 6-4% and plasma prolactin concentration was 2-5 x lower than that found in a random-bred strain with a tumour-induction rate of 41-6%. No difference was found between the 2 strains in the level of either ovarian hormone. It is concluded that the difference between these strains in mammary gland susceptibility to DMBA may be related to plasma prolactin concentration, but it is unlikely to be determined by the ovarian hormones.


*Br. J. Cancer* (1976) **34**, 546

### PLASMA HORMONE LEVELS AND THE INCIDENCE OF CARCINOGEN-INDUCED MAMMARY TUMOURS IN TWO STRAINS OF RAT

R. A. HAWKINS, D. DREWITT, B. FREEDMAN, E. KILLIN, D. A. JENNER* AND E. H. D. CAMERON*

*From the Department of Clinical Surgery, Edinburgh University and *Regional Hormone Laboratory, Edinburgh*

Received 10 May 1976 Accepted 8 June 1976

**Summary.—The incidence of mammary tumours developing after administration of the carcinogen DMBA (at 50 days of age) has been determined in 2 strains of Sprague-Dawley rat. Untreated animals of each strain were exsanguinated in dioestrus at a time corresponding to the early post-carcinogen stage (at 70 days of age) and the plasma concentrations of prolactin, oestradiol-17B and progesterone were measured by radioimmunoassay. In an inbred strain of rats, tumour-induction rate was 6·4% and plasma prolactin concentration was 2·5 × lower than that found in a random-bred strain with a tumour-induction rate of 41·6%. No difference was found between the 2 strains in the level of either ovarian hormone. It is concluded that the difference between these strains in mammary gland susceptibility to DMBA may be related to plasma prolactin concentration, but it is unlikely to be determined by the ovarian hormones.**

In the rodent, mammary tumours can be readily induced following the administration of 7,12-dimethyl benz(a)-anthracene or " DMBA " (Huggins, Grand and Brillantes, 1961) and the sensitivity of the mammary gland to the carcinogen is dependent upon the prevailing endocrine status. In particular, prolactin (Meites, 1972) and ovarian hormones (Geyer *et al.*, 1953; Huggins, Briziarelli and Sutton, 1959; Jabara and Harcourt, 1970) have been reported to alter the sensitivity of their target organ, the mammary gland, to tumorigenesis.

In a previous study from this laboratory (Boyns *et al.*, 1973), it has been shown that in 2 different strains of Sprague-Dawley rat, the rate of tumour induction was much higher in the strain with the higher concentration of plasma prolactin at dioestrus. In view of uncertainty as to whether prolactin or an ovarian hormone has a dominant role in determining the rates of growth and induction of these tumours, we have repeated and extended this work to include assessment of the ovarian hormones, as well as of prolactin, in relation to tumour incidence.

### MATERIALS AND METHODS

*Animals.*—The random-bred strain of rats (OLAC) was originally purchased from Oxford Laboratories. Fresh stock animals have recently been introduced into this strain from Oxford and this appears to have restored sensitivity to tumour induction, which was diminishing with time, to former high levels. The inbred strain of rats (ADRA) was originally purchased from the Animal Diseases Research Association, Edinburgh.

*Tumour induction.*—Two groups of rats (101 OLAC and 62 ADRA) were each treated by the intragastric administration of 30 mg DMBA, at 50–55 days of age. At this age, the inbred rats (ADRA) had a body weight significantly lower (by approximately 15%) than that of the random-bred rats (OLAC), and thus received a slightly higher dose of DMBA/g body wt. Subsequently, animals were palpated for tumours at regular intervals until approximately 6 months of age.

*Collection of blood from animals in dioestrus.*—Vaginal smears were taken daily from 13 OLAC rats and 12 ADRA animals, at approximately 70 days of age. After at least 2 oestrous cycles, rats were killed between 11.30 and 12.03 h, in dioestrus, by exsanguination through the abdominal aorta under ether anaesthesia, the blood being collected into a heparinized syringe. The OLAC rats all exhibited regular 4-day cycles, but the ADRA strain showed longer, irregular cycles as judged from the vaginal smears, with a tendency to show no obvious pro-estrous smear, and a prolongation of the oestrous phase. Accordingly, " dioestrus " was defined for *all* rats as the third day of sequence in which clear, characteristic smears were seen as follows: Day 1: oestrus, Day 2: metoestrus and Day 3: dioestrus (this therefore corresponded to the day before proestrus in the OLAC rats, but such was not necessarily the case in the ADRA animals). Blood was stored on ice and the plasma was separated within 30 min by centrifugation.

*Determination of plasma hormone levels.*—Plasma oestradiol-17B concentration was measured by radioimmunoassay, as previously described, using a single 3·0-ml portion of plasma (Hawkins *et al.*, 1975). Assay sensitivity for this volume of plasma was 0·16 ng/100 ml plasma.

Plasma prolactin concentration was measured also by radioimmunoassay by the method described previously, with minor modifications (Hawkins *et al.*, 1975). [$^{125}$I] iodo-prolactin was prepared by the method of Redshaw and Lynch (1974) and the mean assay sensitivity using such preparations was 3·3 ng RP–1/ml ($n = 22$ assays, range 1 to 7·6 ng/ml). Inevitable deterioration of prolactin standard occurs on storage (NIAMD instructions) and a correction has been made for this using the quality control data, a procedure which results in values lower than those previously reported by us.

Plasma progesterone concentration was measured using duplicate 100-$\mu$l portions of plasma by the method of Cameron and Scarisbrick (1973). Assay sensitivity (defined as the mass required to cause a 10% fall from the maximum found for the standard curve, corrected for losses on extraction) was 0·6 ng/ml plasma.

### RESULTS

The rate of induction of mammary tumours was much greater in the random-bred strain of rats than in the inbred ADRA strain (Table I). Although the tumours were not all examined histologically, in our experience the formation of fibromata generally occurs much later than 4 months after DMBA administration, and thus this difference almost certainly reflects the difference in induction of adenocarcinomata.

TABLE I.—*Incidence of Mammary Tumour Formation in 2 Strains of Rat*

| | OLAC | ADRA |
|---|---|---|
| Total no. treated | 101 | 62 |
| Died due to infections etc. | 12 | 5 |
| Number with tumours | 42 | 4 |
| Number without tumours | 47 | 53 |
| % Incidence | 41·6 | 6·4 |

All rats received 30 mg DMBA intragastrically at 50–55 days of age and were palpated at intervals until approximately 6 months of age. All palpable tumours > 0·5 cm in diameter were counted, irrespective of histology. Incidence was calculated as a percentage of all the rats treated.

TABLE II.—*Plasma Hormone Levels in 2 Strains of Sprague-Dawley Rat*

| Hormone (plasma concentration* units) | Assay sensitivity | Strain of rat: ADRA | Strain of rat: OLAC |
|---|---|---|---|
| Oestradiol-17B (ng/100 ml) | 0·16 | 1·51 ± 1·01 | 1·45 ± 0·68 |
| Progesterone (ng/ml) | 0·6 | 20·7 ± 9·4 | 20·8 ± 11·3 |
| Prolactin (ng RP-1/ml) | 3·3 | 10·2 ± 11·4 | 26·3† ± 17·5 |
| (no animals) | | (12) | (13) |

* Values are means ± s.d.

† Significantly different from the value in the ADRA strain by the Wilcoxon Rank test, $P < 0{\cdot}01$.

Examination of the plasma hormone concentration in these 2 strains of rat (Table II) revealed no significant difference between the animals in the levels of circulating ovarian hormones, but demonstrated the existence of a significant difference in the levels of circulating plasma prolactin.

### DISCUSSION

In an earlier study (Boyns *et al.*, 1973), it was found that in 3 strains of rat, the differences in incidence of tumour induction by DMBA were apparently associated with differences in circulating prolactin concentration measured at 50 days of age, the time of administration of the carcinogen. In the present work, the marked difference in tumour induction rate between the 2 strains of Sprague-Dawley rat has been confirmed, and measurement of the plasma hormone concentrations at 70 days of age shows that the difference in plasma prolactin levels between the strains is maintained from 50 to 70 days. This time would correspond to the early post-carcinogen period in rats treated with DMBA, and in view of the conclusion of Brown and Shellabarger (1974) that differences in tumour susceptibility to DMBA are determined *after* carcinogen-cell interaction, it may well be the time at which inter-strain differences are involved. In contrast to the difference between strains in plasma prolactin concentration at this stage, we found no difference in the plasma concentration of either of 2 major ovarian hormones. A similar, apparent association between increased incidence of tumour induction and increased plasma prolactin levels has been noted in rats fed on a high fat diet (Chan, Didato and Cohen, 1975) but in that study the ovarian hormones were not examined.

The effects of prolactin and ovarian hormones on the induction of mammary tumours by carcinogens are complex. The absence of either the pituitary gland (Foulds, 1975) or the ovary (Dao, 1962; Talwalker, Meites and Mizuno, 1964) has been reported to inhibit tumour induction. Thus both prolactin (Meites, 1972) and oestrogen (Sinha and Dao, 1972) have been implicated in tumorigenesis, though tumours can be induced in ovariectomized rats treated by administration of prolactin and growth hormone (Talwalker *et al.*, 1964). By contrast, high levels of either prolactin (Meites, 1972; Gala and Loginsky, 1973) or oestrogen (Nagasawa *et al.*, 1974) may protect the mammary gland from carcinogenesis.

In the 2 strains of rat studied here it seems unlikely that the level of either of the 2 major ovarian hormones normally determines the susceptibility of the mammary gland to carcinogenesis.

It should be noted, however, that in the mouse, attempts to relate plasma, pituitary or urinary levels of prolactin to the incidence of spontaneous tumours in 2 different strains have led to the suggestion that, for prolactin, mammary turnover rather than plasma level may determine susceptibility to spontaneous mammary tumorigenesis (Sinha, Selby and Vanderlaan, 1974; Sinha *et al.*, 1974).

We wish to thank Professor A. P. M. Forrest for his advice and encouragement, and Mrs Dorothy Gray for expert technical assistance. Mr B. Freedman and Mrs Gray are grateful to the Cancer Research Campaign (Grant No. SP 1256 to Professor Forrest) for support.

We thank the National Institutes of Health, Bethesda, Maryland, as the sole source of reagents for the radioimmunoassay of rat prolactin.

### REFERENCES

BOYNS, A. R., BUCHAN, R., COLE, E. N., FORREST, A. P. M. & GRIFFITHS, K. (1973) Basal Prolactin Blood Levels in 3 Strains of Rat with Differing Incidence of 7,12-dimethyl benz(a)anthracene induced Mammary Tumours. *Eur. J. Cancer*, **9**, 169.

BROWN, R. D. & SHELLABARGER, C. J. (1974) Mammary Neoplasia in Sprague-Dawley or Long-Evans Rats after *in vitro* 7,12-dimethyl benz(a)-anthracene. *Cancer Res*, **34**, 2594.

CAMERON, E. H. D. & SCARISBRICK, J. J. (1973) Radioimmunoassay of Plasma Progesterone. *Clin. Chem.*, **19**, 1403.

CHAN, P., DIDATO, F. & COHEN, L. A. (1975) High Dietary Fat, Elevation of Rat Serum Prolactin and Mammary Cancer. *Proc. Soc. exp. Biol. Med.*, **149**, 133.

DAO, T. L. (1962) The Role of Ovarian Hormones in Initiating the Induction of Mammary Cancer in Rats by Polynuclear Hydrocarbons. *Cancer Res.*, **22**, 972.

FOULDS, L. (1975) Neoplastic Development **2**, Chapter 9. In *Mammary Neoplasia in Laboratory Animals.* London: Academic Press.

GALA, R. R. & LOGINSKY, S. J. (1973) Correlation between Serum Prolactin Levels and Incidence of Mammary Tumours Induced by 7,12-dimethyl benz(a)anthracene in the Rat. *J. natn Cancer Inst.*, **51**, 593.

GEYER, R. P., BRYANT, J. E., BLEISCH, V. R., PIERCE, E. M. & STARE, F. J. (1953) Effect of Dose and Hormones on Tumour Production in Rats Given Emulsified 9,10-dimethyl,-1,2-benzanthracene Intravenously *Cancer Res.*, **13**, 503.

HAWKINS, R. A., FREEDMAN, B., MARSHALL, A. & KILLIN, E. (1975) Oestradiol-17B and Prolactin Levels in Rat Peripheral Plasma. *Br. J. Cancer*, **32**, 179.

HUGGINS, C., BRIZIARELLI, G. & SUTTON, H. (1959) Rapid Induction of Mammary Carcinoma in the Rat and the Influence of Hormones on the Tumours. *J. exp. Med.*, **109**, 25.

HUGGINS, C., GRAND, L. S. & BRILLANTES, F. P. (1961) Mammary Cancer Induced by a Single Feeding of Polynuclear Hydrocarbons and its Suppression. *Nature, Lond.*, **189**, 204.

JABARA, A. G. & HARCOURT, A. G. (1970) Effect of Progesterone and Ovariectomy on Mammary Tumours Induced by 7,12-dimethyl benz(a)-anthracene in Sprague-Dawley Rats. *Pathology*, **2**, 115.

MEITES, J. (1972) Relation of Prolactin to Mammary Tumorigenesis and Growth in Rats. In *Prolactin and Carcinogenesis* Eds. A. R. Boyns & K. Griffiths. Cardiff: Alpha Omega Alpha.

NAGASAWA, H., YANAI, R., SHODANO, M., NAKAMURA, T. & TANABE, Y. (1974) Effect of Neonatally Administered Estrogen or Prolactin on Normal and Neoplastic Mammary Growth and Serum estradiol-17B level in Rats. *Cancer Res.*, **34**, 2643.

REDSHAW, M. R. & LYNCH, S. S. (1974) An Improved Method for the Preparation of Iodinated Antigens for Radioimmunoassay. *J. Endocr.*, **60**, 527.

SINHA, D. & DAO, T. L. (1972) Estrogen and Induction of Mammary Cancer. In *Estrogen Target Tissues and Neoplasia.* Ed. T. L. Dao. Chicago: University of Chicago Press.

SINHA, Y. N., SALOCKS, C. B., LEWIS, U. J. & WANDERLAAN, W. P. (1974) Influence of Nursing, on the Release of Prolactin and GH in Mice with High and Low Incidence of Mammary Tumours. *Endocrinology*, **95**, 947.

SINHA, Y. N., SELBY, F. W. & VANDERLAAN, W. P. (1974) The Natural History of Prolactin and GH Secretion in Mice with High and Low incidence of Mammary-Tumours. *Endocrinology*, **94**, 757.

TALWALKER, P. K., MEITES, J. & MIZUNO, H. (1964) Mammary Tumour Induction by Estrogen on Anterior Pituitary Hormones in Ovariectomised Rats given 7,12-dimethyl-1,2-benzanthracene. *Proc. Soc. exp. Biol. Med.*, **116**, 531.